# Restoration of a functional antiviral immune response to chronic HBV infection by reducing viral antigen load: if not sufficient, is it necessary?

**DOI:** 10.1080/22221751.2021.1952851

**Published:** 2021-08-07

**Authors:** Sisi Yang, Wanjia Zeng, Jiming Zhang, Fengmin Lu, Jinhong Chang, Ju-Tao Guo

**Affiliations:** a Baruch S. Blumberg Institute, Doylestown, PA, USA; b Huashan Hospital, Fudan University, Shanghai, People’s Republic of China; c Peking University Health Science Center, Beijing, People’s Republic of China

**Keywords:** Hepatitis B virus, HBsAg, immune tolerance, immunotherapy, therapeutic vaccination

## Abstract

The prolonged viral antigen stimulation is the driving force for the development of immune tolerance to chronic hepatitis B virus (HBV) infection. The sustained reduction of viral proteins may allow for the recovery and efficient activation of HBV-specific T and B cells by immune-stimulating agents, checkpoint blockades and/or therapeutic vaccinations. Recently, several therapeutic approaches have been shown to significantly reduce intrahepatic viral proteins and/or circulating HBV surface antigen (HBsAg) with variable impacts on the host antiviral immune responses in animal models or human clinical trials. It remains to be further investigated whether reduction of viral protein expression or induction of intrahepatic viral protein degradation is more efficacious to break the immune tolerance to chronic HBV infection. It is also of great interest to know if the accelerated clearance of circulating HBsAg by antibodies has a long-term immunological impact on HBV infection and disease progression. Although it is clear that removal of antigen stimulation alone is not sufficient to induce the functional recovery of exhausted T and B cells, accumulating evidence suggests that the reduction of viral antigen load appears to facilitate the therapeutic activation of functional antiviral immunity in chronic HBV carriers. Based on a systematic review of the findings in animal models and clinical studies, the research directions toward discovery and development of more efficacious therapeutic approaches to reinvigorate HBV-specific adaptive immune function and achieve the durable control of chronic HBV infection, i.e. a functional cure, in the vast majority of treated patients are discussed.

## Introduction

Hepatitis B virus (HBV) chronically infects 257 million people worldwide and causes 800,000 deaths annually due to cirrhosis and hepatocellular carcinoma (HCC). Treatment of chronic hepatitis B (CHB) with nucleos(t)ide analogue (NUC) viral DNA polymerase inhibitors, such as entecavir and tenofovir, alone or in combination with pegylated alpha-interferon (IFN-α), can potently inhibit viral replication and prevent liver disease progression, but fails to induce a durable off-drug control of HBV infection in the majority of treated patients [1]. The reason for the failure of cure is that the currently available antiviral drugs cannot eliminate or inactivate viral covalently closed circular DNA (cccDNA), a nuclear viral replication intermediate that serves as the template for the transcription of viral RNAs. cccDNA is the most stable viral replication intermediate and resource of viral replication rebound after the termination of antiviral therapy [2]. In fact, even the clinical resolution of acute HBV infection is not the complete eradication of HBV cccDNA from the infected individuals, but a constant suppression of residual HBV infection by the immune surveillance of HBV-specific T and B cells [3]. This is evident by the reactivation of HBV replication under immunocompromised conditions even decades after clinical resolution of acute HBV infection [4]. Apparently, although the eradication of cccDNA from the infected individuals, the virological cure, is an ideal therapeutic endpoint, it is difficult to achieve. Instead, the therapeutic goal proposed by the world-leading professional associations, the American Association for the Study of Liver Diseases (AASLD) and the European Association for the Study of Liver Disease (EASL), is to mimic the natural resolution of acute HBV infection by restoration or reconstitution of a functional antiviral immune response to durably control the chronic HBV infection, i.e. the functional cure of CHB, as indicated by the seroclearance of HBsAg, with a finite duration of therapies [5].

## The outcomes and pathogenesis of HBV infection are determined by host antiviral immune responses

Unlike many other viruses that activate vigorous host cellular responses and kill the infected cells, HBV is a stealthy, non-cytopathic virus that does not activate a detectable “conventional” innate immune response and cellular transcriptome change in infected hepatocytes [6–8]. HBV infection of humans can be either resolved within six months (transient/acute) or persistent (chronic) for decades. The acute infection can be asymptomatic or causes hepatitis, which is generally mild and self-limited, but occasionally life-threatening fulminant hepatitis. Chronic HBV infection usually experiences four clinical stages: the initial phase of high viral load with no liver disease (immune tolerant or non-inflammatory phase) to active liver disease stage with HBeAg to anti-HBe seroconversion (immune clearance phase), followed by the inactive phase with a low viral replication and minimal liver diseases. Some of the inactive HBV carriers may revert to an immune reactivation phase with recurrent liver diseases and increased viral load years later [9].

Because HBV infection of hepatocytes does not directly induce cytopathic effects, the outcomes and pathogenesis of HBV infection are primarily determined by the nature and strength of host antiviral adaptive immune responses. Specifically, clinical studies showed that the resolution of acute HBV infection is associated with vigorous polyclonal CD8+ cytotoxic T lymphocyte (CTL) response and induction of antibodies against viral surface antigen [10]. Mechanistically, in addition to the killing of infected hepatocytes, CTLs also non-cytolytically cure the viral infected hepatocytes by cytokine-induced antiviral responses that suppress viral replication and eliminate/inactivate cccDNA [11,12]. Antibodies against HBsAg become detectable after the clearance of circulating HBsAg and work together with HBV-specific CD4+ and CD8+ memory T cells to control the residual HBV after apparent clinical resolution of an acute HBV infection over decades. Pathologically, the necrotic liver inflammation, i.e. hepatitis, during HBV infection is considered as the consequence of adaptive cellular immune responses, particularly CTLs, against HBV-infected hepatocytes [11]. Obviously, the balance between non-cytopathic and cytopathic effects of HBV-specific CD8+ T cells as well as the dynamics of immune response-induced hepatocyte killing/regeneration are critical in mediating HBV control without massive liver damage [13]. However, a recent study in HBV-infected chimpanzees indicates that the humoral immune response against viral proteins, particularly core protein, may play a critical role in the pathogenesis of severe or fulminant hepatitis [14].

**Figure 1:**
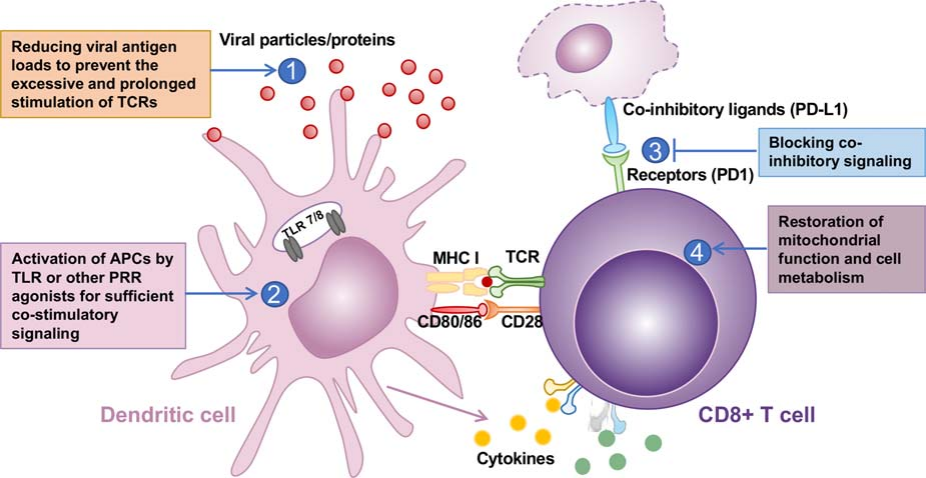
.#Therapeutic strategies to restore the functional T cell immune response. Activation of CD8+ T cells requires MHC-I presentation of antigenic peptide to TCR and co-stimulatory (such as CD80/CD86-CD28 engagement) and cytokine signaling. Excessive TCR stimulation induces the expression of co-inhibitory receptors on T cells. Activation of co-inhibitory receptors induces inhibitory gene expression and functional exhaustion of T cells. Mitochondrial dysfunction and extensive reprogramming of cell metabolism also contribute to the functional exhaustion of T cells. Four therapeutics strategies for restoration of exhausted CD8+ T cell are proposed.

Compared to those with acute infection, patients with chronic HBV infection are associated with the functional exhaustion or depletion of viral antigen-specific T cells [10,15] and atypical HBsAg-specific memory B cells deficient in the production of antibodies [16,17]. The immune tolerance or exhaustion of HBV-specific T and B cells is induced by continued stimulation of viral antigens in the setting of persistent viral replication. However, the mechanisms underlying the dysfunction of HBV-specific T and B cells are still not completely understood. It had been shown that although HBV-specific CD4+ and CD8+ T cells responses are initially induced, the persistence of viral antigens drives the differentiation of activated T cells to a functionally exhausted stage deficient in cytokine production, cytotoxicity and proliferative capacities [18,19]. The excessive antigen stimulation not only induces epigenetic reprogramming of CD8+ T cell gene regulation that reduce the expression of cytokines and effector molecules, but also induce the expression of multiple inhibitory receptors, such as programmed cell death protein (PD1) and cytotoxic T-lymphocyte-associated protein 4 (CTLA-4) [20,21] (Figure 1). Meanwhile, over-expression of several checkpoint receptor ligands, such as PD-L1 and galectin-9, has been observed on circulating and intrahepatic antigen-presenting cells and on liver resident Kupffer cells (KCs), respectively [22]. The interaction of these co-inhibitory receptors with their respective ligands inhibits the signal transduction of T-cell receptor (TCR) and co-stimulatory receptors and induces the expression of inhibitory genes [23]. Recently, it was reported that the thymocyte selection-associated high mobility group-box protein (Tox) plays a critical role in the development and maintenance of exhausted T lymphocytes [24]. Cell biological analyses also revealed that the exhausted T cells displayed mitochondrial dysfunction and metabolic reprogramming [25] and are more prone to apoptosis mediated by the up-regulation of the death receptor TRAIL-2 and the pro-apoptotic mediator BIM [26,27]. Although the excessive viral antigen is the primary driver of T and B cells exhaustion, dysfunction of antigen presentation cells, limited CD4+ T cell help, suppression by regulatory T cells, myeloid-derived suppressor cells (MDSC) and immunosuppressive cytokines such as IL-10 and transforming growth factor β (TGF-β) also favour the induction of T and B cell exhaustion [19,28,29].

## Therapeutic strategies to restore a functional adaptive immune response in chronic hepatitis B patients

Understanding the mechanism of HBV-specific T and B cell exhaustion is the basis for the development of therapeutics to restore the functions of exhausted cells. As illustrated in Figure 1, it has been demonstrated that disrupting the interaction of co-inhibitory receptors with their ligands, i.e. checkpoint blockade, can significantly reinvigorate the cytokine production and proliferation potential of exhausted T cells *in vitro* [18,21,30,31]. A phase 1b clinical trial of PD1 blockade with nivolumab in HBeAg negative chronically HBV-infected patients showed a modest decline of HBsAg in most of the patients, while one patient achieved a sustained HBsAg seroclearance [16,32]. In addition to the checkpoint blockade therapies, studies in transgenic mice also demonstrated that activation of CD40-mediated co-stimulatory pathway rescued HBV-specific CD8+ T cells from PD-1-mediated exhaustion in HBV transgenic mice [33]. Moreover, improvement of mitochondrial function and correction of impaired metabolism of exhausted T cells, stimulation of antigen presentation cells (APCs) with the ligands of pattern recognition receptors (PRRs) and inhibition of Treg functions also partially restore the cytotoxicity and cytokine production capacities of exhausted HBV specific CD8+ T cells *in vitro* or *in vivo* [21,30,34]. However, the exhausted T cells are phenotypically heterogeneous and epigenetically reprogrammed extensively [35–37]. It is unlikely that the immunological functions of exhausted T and B cells can be completely restored by targeting a single molecular pathway.

Because of the critical role of excessive viral antigen stimulation in the induction of T and B cell exhaustion, reducing the viral proteins in hepatocytes and/or viral antigen loads in circulation, including HBsAg and HBeAg, had been considered as a prerequisite for the functional recovery of exhausted immune cells in CHB patients (Figure 1). In support of this hypothesis, it was reported that the functionality of HBV-specific T cells in CHB patients inversely correlates to the levels of HBsAg antigenemia and viral loads [38,39]. In addition, although the long-term therapies with nucleos(t)ide analogues do not generally lower serum HBsAg levels, a partial recovery of HBV-specific T cell function had been observed in patients receiving the therapies [40]. A plausible explanation for this finding is the significant reduction of viral proteins other than HBsAg, due to the significant reduction of cccDNA after the long-term suppression of viral DNA replication [41]. Moreover, pegylated IFN-α treatment of HBeAg- negative CHB patients did not induce the functional recovery of exhausted HBV-specific CD8 T cell response, but the decrease of viral loads was associated with an increase in the frequency of circulating CD56bright NK cells, increased expression of the activatory receptor NKp46 and the cytotoxic receptor TRAIL by NK cells and recovery of their IFN-γ production [42]. However, the frequency and function of HBsAg-specific B cells are partially restored in CHB patients upon pegylated IFN-α treatment-induced loss of HBsAg and anti-HBs seroconversion [43]. In addition, the functional cure rate of pegylated IFN-α treatment is significantly higher in patients with lower levels of HBsAg [44]. It is thus conceivable that the prolonged reduction of viral proteins may allow for a more profound recovery of HBV-specific T and B cells. However, clinical studies in CHB patients treated with drugs that are capable of reducing one or multiple viral proteins are required to directly prove the concept of whether reduction of viral antigen production or acceleration of viral protein degradation improves the function of HBV-specific T and/or B cells. It is also important to determine the viral antigens and the magnitudes of their reduction required for the restoration of T cell functions. Currently, three therapeutic modalities, including small interference RNA (siRNA) [45], antisense oligonucleotides (ASOs) [46] and small molecular drugs that induce viral RNA degradation [47], have been demonstrated in animal models or human clinical trials to significantly reduce the expression of multiple viral proteins and suppress viral replication. In addition, inhibitors of HBsAg secretion and monoclonal antibodies against HBsAg that can reduce circulating HBsAg are also under preclinical and clinical development. These clinical studies provide a unique opportunity to evaluate the impact of reducing intrahepatic and/or circulating HBV proteins on the functional status of HBV-specific T and B cells and their responses to immunothereapeutics. It is our hope that the knowledge, including the success and failure, learned from these clinical trials will ultimately help the discovery of feasible therapeutic approaches to restore a functional antiviral immune response for the functional cure of CHB.

## Effects of reducing intrahepatic viral protein expression and/or circulating HBsAg on antiviral immune responses in mouse models

HBV transgenic mice lineage 1.3.32 contain a single copy of the terminally redundant 1.3mer HBV ayw genome integrated into the mouse chromosomal DNA [48]. HBV RNA transcription turns on progressively during the early postnatal liver development through gradual demethylation of the integrated viral genomic DNA and reaches maximal level at approximately 4 weeks after birth [49]. The early postnatal activation of HBV replication in the hepatocytes mimics neonate infection of HBV, which attributes to the most majority of chronic HBV infection in humans. Therefore, this transgenic mouse line had been extensively used in studying the mechanism of HBV-specific immune tolerance and immune control of HBV infection [50]. In addition, persistent replication of HBV in mice can also be established by transduction of HBV replicons *via* recombinant adenoviruses [51] or adenovirus-associated viruses (AAV) [52] as well as hydrodynamic injection of plasmid-based HBV replicons or recombinant cccDNA-like molecules [53]. Although these mouse models do not support dynamic HBV infection/dissemination and viral RNA transcription is not from authentic cccDNA, they provide convenient immune-competent small animal models to study host immune response to HBV infection.

In order to determine the role of circulating and intrahepatic HBsAg in the induction of CD8+ T cell exhaustion, Fumagalli and colleagues reported recently that serum HBsAg clearance induced by either spontaneous seroconversion or therapeutic monoclonal antibodies has only a minimal effect on the expansion of HBV-specific naive CD8+ T cells undergoing intrahepatic priming [54]. Interestingly, another study by this research group with transgenic mice expressing high level of HBcAg in almost all the hepatocytes and mice expressing lower level of HBcAg in approximately 10% of hepatocytes by adenoviral transduction showed that adoptively transferred HBcAg-specific naïve CD8+ T cells failed to differentiate into effector CTLs in mice expressing either high or low levels of HBcAg in hepatocytes [50]. However, the adoptively transferred HBcAg-specific naïve CD8+ T cells efficiently develop into effectors CTLs in mice transduced with recombinant replication-defective lymphocytic choriomeningitis virus (LCMV)-based vectors expressing HBV core protein in Kupffer cells (KCs) and hepatic dendritic cells (DCs) [50]. Because HBcAg cannot be secreted into circulation, these findings again clearly indicate that expression of viral antigen in hepatocytes is tolerogenic due to the lack of sufficient costimulatory signals and reduction of HBcAg expression in hepatocytes by more than 10 folds cannot prevent the induction of CD8+ T cell tolerance. However, it was found that treatment of HBV transgenic mice with type I IFN or IL-2 can rescue, at least in part, the development of the adoptively transferred HBV-specific naïve CD8+ T cells to functional effectors CTLs [50, 55]. These findings imply that IFN-α or pattern recognition receptor (PRR) agonist therapy of CHB may activate host antiviral immune response *via* partially rescuing the functional development of hepatocyte-primed CTLs (Figure 1).

However, a recent report by Michler et al showed that reduction of HBV viral protein expression by siRNA, but not the inhibition of viral replication by entecavir treatment, in HBV transgenic mice or AAV-HBV transduced mice enhanced the activation of polyclonal HBV-specific CD8+ effectors T cells and induction of neutralizing antibodies by a therapeutic vaccination regimen for durable control of HBV replication [56]. In addition, depletion of circulating HBsAg from AAV-HBV transduced mice by a monoclonal anti-HBsAg antibody enabled the activation of HBsAg-specific CD4+ T cells and induction of neutralizing antibody by subsequent Engerix-B vaccination. Moreover, TLR9 agonist-adjuvanted Engerix-B vaccination induced the clearance of HBV in both serum and liver [57]. These studies indicated that although the clearance of circulating HBsAg and reduction of hepatic viral protein by more than 10 fold cannot restore the functional development of hepatocyte-primed CD8+ T cells, it does allow the activation of antiviral adaptive immune responses by vaccination for efficient control of HBV infection in mice.

## Breaking the immune tolerance to chronic WHV infection in woodchucks

Similar to HBV infection of humans, adulthood woodchuck hepatitis virus (WHV) infection is generally transient and resolved within 24 weeks, whereas congenital and neonate WHV infection usually results in life-long persistent infection and development of hepatocellular carcinoma in almost 100% of infected woodchucks within 3 years [58]. Woodchuck model had been used extensively to investigate the mechanism of immunological resolution of acute HBV infection and evaluate therapeutics for the cure of chronic HBV infection. Particularly, the demonstration of sustained suppression of viral replication and WHsAg to anti-WHs seroconversion with the TLR-7 agonist GS-9620 in a subset of WHV chronically infected woodchucks ignited the hope for the functional cure of CHB by PRR agonist therapy [59]. However, the following studies in chimpanzees and clinical trials showed limited efficacy of GS-9620 in tolerable dosing conditions [60,61]. Recently, it was discovered that a small molecular inhibitor (RG7834) of host cellular noncanonical poly(A) polymerases 5 and 7 (PAPD5 and PAPD7) can induce HBV mRNA degradation and subsequently inhibit viral replication and expression/secretion of multiple viral proteins [62]. Treatment of chronically WHV-infected woodchucks with RG7834 alone or in combination with entecavir and pegylated woodchuck IFN-α for 14 weeks reduced serum WHsAg by 2.57 or 5 log10, and WHV DNA by 1.71 or 7.45 log10 from baseline, respectively [63]. However, after termination of therapy, both WHsAg and WHV DNA levels rebounded, suggesting that such dramatic reductions of intrahepatic viral protein expression and circulating WHsAg are not sufficient to restore a functional antiviral immune response for the durable control of viral infection. It will be interesting to determine in this animal model with these therapeutic regimens the extents and durations of viral protein expression reduction that are required for the activation of a functional antiviral immune response by the variety of therapeutic vaccination strategies. It is also interesting to test whether reduction of viral antigen load will potentiate the therapeutic efficacy of TLR7/8 agonists.

## Restoration of functional immune response in CHB patients

**Figure 2.**
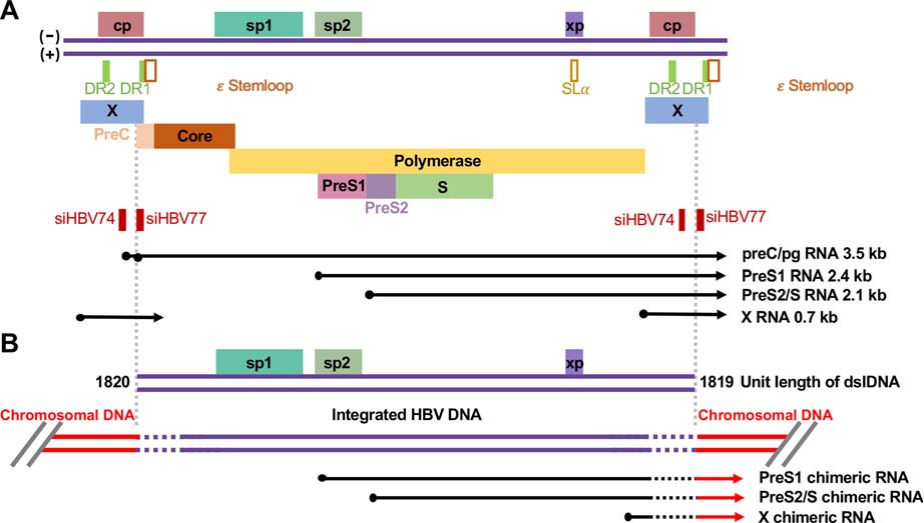
Transcription maps of cccDNA and integrated HBV DNA. Schematic presentation of HBV RNA transcribed from cccDNA (A) and integrated HBV DNA (B). For the convenience of illustration, cccDNA transcription template is presented as a 1.3mer liner genome. Four promoters (cp: core promoter; sp1: preS1 promoter; sp2: preS2/S promoter and xp: HBx promoter). Epsilon (ϵ) RNA element, RNA stem loop (SLα), direct repeat sequences 1 and 2 (DR1 and DR2) are presented. Double stranded linear (dsl) DNA is synthesized by in situ priming of plus-stranded DNA and is the primary precursor of integrated viral DNA. Due to the error-prone end processing during the integration via non-homologous end joining DNA repair pathway, deletions and insertions occur in the terminal regions of viral DNA (dashed lines). Due to the loss of 5’-terminal core promoter, the integrated DNA cannot transcribe preC/pgRNA. Because of the loss of the authentic viral poly A adenylation signal at the 3’ terminus, the RNA transcription initiated from sp1, sp2 and xp will extended into host chromosomal DNA and terminated at the encountered poly A adenylation signal. Therefore, all the viral RNA transcripts from integrated DNA are HBV-host cellular chimeras. The binding sites of two siRNA in ARC-520 are indicated.

Targeted degradation of HBV mRNA by RNA interference (RNAi) or antisense oligonucleotides (ASOs) have been achieved not only in animal models but also in humans in clinical trials. Due to the extensive sequence overlaps of HBV RNA transcripts, a single siRNA or ASO targets multiple or all the viral transcripts (Figure 2). The first RNAi-based drug in clinical trials is ARC-520 (Arrowhead Research Corporation, Pasadena, California, USA), containing a hepatocyte targeted, reversibly masked membrane-active peptide (NAG-MLP) to drive endosomal release of two synthetic RNAi triggers (cholesterol-conjugated siRNAs, siHBV-74 and siHBV-77) which target at HBV X region [64]. A single dosing of ARC-520 reduced serum HBsAg by 1.4 ±0.1 log10 in NUC-naïve HBeAg positive patients, but only reduced serum HBsAg by approximately 0.3 log10 in NUC-naïve HBeAg negative patients. Further studies revealed that the primary RNA transcripts for HBsAg synthesis are derived from integrated HBV DNA, but not cccDNA, in NUC experienced and NUC-naive HBeAg negative CHB patients and chimpanzees [65]. Due to the lack of the targets of siHBV-74 and siHBV-77 in the RNA transcripts derived from integrated DNA (Figure 2), ARC-520 is much less efficacious in HBeAg negative CHB patients and chimpanzees [65]. As expected, a second-generation siRNA (ARC-521) targeting all HBV transcripts from both cccDNA and integrated DNA significantly reduced HBsAg in HBeAg negative CHB patients [65]. Currently, a new generation of GalNAc-conjugated siRNAs targeting all HBV transcripts, such as JNJ-3989 and AB-729, are well-tolerated in patients with CHB and reduce HBsAg more than 1 log10 IU/mL after a single dosing. Similarly, GalNAc-conjugated ASOs, GSK3389404, is well tolerated to repeated dosing up to 120 mg once a week for 4 weeks in a phase 1 clinical trial [66]. A Phase 2a trial with GSK3228836 showed significant and prolonged reduction of HBV antigenemia after four weeks treatment of CHB patients under NUC treatment or without NUC therapy. It is particularly interesting that GSK3228836 treatment induced more significant reduction and even anti-HBs seroconversion in a portion of NUC-naïve HBeAg-negative, but not HBeAg positive, patients. Asymptomatic and self-resolved ALT flares occurred during HBsAg clearance. While these proof-of-concept studies validated the antiviral efficacy of liver-targeting siRNA and ASOs, the immunological response of HBsAg reduction in patients have not been investigated.

In addition to the HBV RNA-targeting oligonucleotide therapeutics discussed above, it was found that nucleic acid polymers (NAPs), single-strand phosphorothioate sequence non-specific single-stranded DNA of minimal 40 nucleotides in length, demonstrated superior efficacy to induce the functional cure of chronic hepadnaviral infection in ducks and in human clinical trials [67]. Ducks with persistent duck hepatitis B virus (DHBV) infection after 28 days of REP 2055 treatment lead to initial rapid reductions in serum DHBsAg and DHBV DNA and induction of anti-DHBs antibodies in all ducks, and 6 out of 11 ducks experienced a sustained virologic response at 9 or 16 weeks of follow-up [68]. The clearance of DHBsAg from serum was companied with the significant reduction of HBsAg-positive hepatocytes in the liver. NAPs inhibition of HBsAg secretion in human hepatoma-derived HBV replicating cells (HepG2.2.15) had been demonstrated under the condition of co-treatment with a small molecular compound UNC7938 that promotes the endosomal escaping of NAPs [69]. In a recent phase 2 clinical trial, one of the two forms of NAPs, REP 2139 or REP 2165, was added to CHB patients under TDF and pegylated IFN-α combination therapy for 24 weeks and treated for additional 48 weeks and followed for 24 weeks after the termination of the triple combination therapy [70]. No severe adverse events were reported in patients with NAPs. However, elevated ALT levels were observed during NAP therapy, which was correlated with initial decrease of HBsAg and normalized during therapy and follow-up. At the end of therapy, 24 out of 40 patients achieved the loss of serum HBsAg and serum conversion to anti-HBs. At the end of 24 weeks of follow-up, 14 out of 34 patients remained the negativity of serum HBsAg and can be considered as a “functional” cure [70].

While it is rather intriguing that the combination of standard-of-care CHB therapeutics with NAPs achieved a functional cure in more than 40% of treated patients, the therapeutic efficacy of NAP-based combination therapy requires further validation in expanded clinical trials. It also remains to know whether the improved therapeutic efficacy is due to the inhibition of HBsAg secretion by the NAPs. Apparently, uncovering the immunological mechanism of NAP combination therapy-induced HBsAg seroclearance and seroconversion may reveal the molecular targets for more efficient restoration of functional immune response against HBV in CHB patients and facilitate the improvement of NAPs or discovery of novel therapeutics to functionally cure CHB in a much larger patient population.

## Therapeutic potential of monoclonal antibodies for CHB

**Figure 3.**
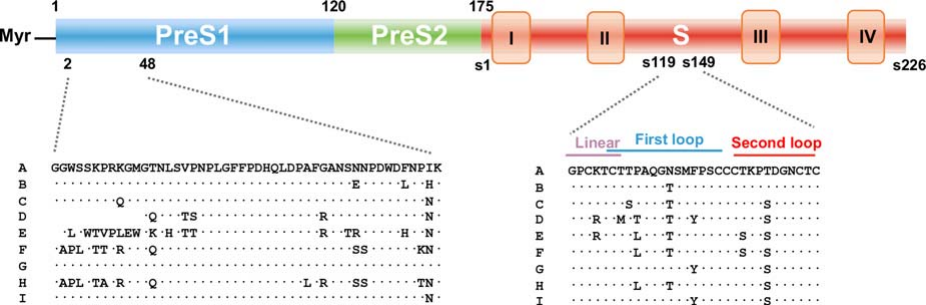
Distribution of neutralizing epitopes in HBV envelope proteins. The epitopes recognized by human neutralization antibodies against HBV are mapped at the N-terminal NTCP binding region of pre-S1 and antigenic loop of S region. Sequences of these epitopes from Genotype A to I were aligned.

Humoral immune response to HBV envelop proteins, collectively measured as antibodies against HBsAg, or anti-HBs, becomes detectable after clinical resolution of acute or chronic HBV infection and successful HBV vaccination. Although its role in the resolution of HBV infection remains to be firmly established, the antibody response plays essential roles in the prevention of HBV infection and control of the residual HBV replication after apparent clinical resolution of HBV infection. The tremendous success of antibody drugs in many therapeutic areas and technical development in HBV infection cell cultures and animal models empower the development of monoclonal antibodies targeting HBV envelope proteins as a key therapeutic modality for the management of CHB [71]. As illustrated in Figure 3, many monoclonal antibodies targeting the epitopes located at the N-terminal sodium/taurocholate cotransporting polypeptide (NTCP) receptor binding region in pre-S and antigenic loop of S region have been isolated from B cells derived from individuals who resolved a natural HBV infection or successfully responded to HBV vaccination. These antibodies demonstrated pan-genotype (broadly) or genotype-specific neutralizing activity to HBV infection in HBV infection cell culture assays.

Therapeutically, broadly neutralizing HBV antibodies can block the spread of viral infection and accelerate the clearance of HBV virions and subviral particles from extracellular space. Interestingly, a study showed that a combination of two human monoclonal anti-HBs (HepeX-B) antibodies can also inhibit the release of HBV viral particles from infected cells [72]. The antibodies can additionally recognize viral envelope proteins expressing on the surface of infected hepatocytes and kill the infected cells by natural killer cell-mediated antibody-dependent cellular cytotoxicity (ADCC) or complement-dependent cytotoxicity (CDC). Unfortunately, viral envelope proteins are located primarily at intracellular membranes and not normally expressed in the plasma membrane of hepatocytes at a significant amount, this latter antiviral mechanism should thus not significantly contribute to the therapeutic efficacy of antibody therapies to CHB. In agreement with their immune effector functions, several studies have demonstrated that administration of neutralizing HBV antibodies in multiple HBV-persistent infection mouse models significantly reduced or depleted HBV virions and subviral particles in blood [73–76]. In particular, depletion of circulating HBsAg in AAV-HBV transduced mice enabled the induction of HBV-specific B and T cell immune responses by distinct vaccination strategies [57,77]. Clinically, several human monoclonal antibodies against HBsAg had been shown to significantly reduce viral load and HBsAg antigenemia [72,78]. However, the long-term therapeutic efficacy, particularly in combination with other antiviral agents and immunostimulatory drugs, remains to be evaluated. Interestingly, a recent study showed that engineered anti-influenza virus IgG with enhanced activity to bind a subset of Fc receptor, FcγRIIa, on dendritic cells stimulates dendritic cell maturation and induces protective CD8+ T cell responses to the virus [79]. This finding implies that the broadly neutralizing HBV antibodies may be genetically modified to activate specific dendritic cell and T cell pathway for efficient induction of HBV-specific cellular immune response to durably control the chronic HBV infection.

## Summary and perspectives

Although the eradication of HBV cccDNA and/or all the infected hepatocytes to achieve a virological cure of CHB is not entirely impossible, the realistic goal of CHB therapy is the durable off-drug control of residual HBV infection and stop/reverse the liver disease progression, i.e. the functional cure. The biomarker for the functional cure of CHB is the loss of circulating HBsAg. Obviously, unlike the virological cure, the functional cure relies on the constant immune surveillance and control of residue HBV infection. Therefore, restoration or re-establishment of a functional HBV-specific T and B lymphocyte-mediated immune response in the CHB patients is essential to achieve the functional cure. Considering the essential role of viral proteins in driving the differentiation of activated T and B cells toward functional exhaustion, reduction of viral protein expression and elimination of circulating HBsAg and HBeAg is considered as the basis for the reinvigoration of HBV-specific T and B cell immunity. Apparently, due to the extensive metabolic and epigenetic reprogramming of HBV-specific T and B cells in sequential differentiation toward the different degrees of exhaustion status during the long-term chronic infection, it is not surprising that reducing or eliminating HBV antigen stimulation alone is insufficient for the functional recovery of the exhausted T and B cells [80]. However, studies in the various animal models convincingly demonstrated that reduction of intrahepatic and/or circulating HBV antigens does improve the responsiveness of HBV-specific T and B cells to therapeutic vaccination. It will be interesting to further investigate whether the reduced viral antigen load and/or circulating HBsAg and HBeAg also potentiate the therapeutic efficacy of immunostimulating therapy with interferons, PRR agonists and checkpoint blockades. With the advent of therapeutics that can reduce viral protein expression *via* distinct mechanisms, induce viral protein degradation or accelerate the clearance of circulating HBsAg as well as the development of various novel vaccination strategies and immune-stimulating drugs in next a few years, the best combination therapeutic strategies of viral antigen load reducers and immunostimulators for efficient and safe immune control of chronic HBV infection will likely be discovered.

## References

[1] Liang TJ, Block TM, McMahon BJ, et al. Present and future therapies of hepatitis B: from discovery to cure. Hepatology. 2015 Dec;62(6):1893–1908. 26239691 10.1002/hep.28025 PMC4681668

[2] Hu J, Cheng J, Tang L, et al. Virological basis for the cure of chronic hepatitis B. ACS Infect Dis. 2019 May 10;5(5):659–674. 29893548 10.1021/acsinfecdis.8b00081 PMC8026331

[3] Rehermann B, Ferrari C, Pasquinelli C, et al. The hepatitis B virus persists for decades after patients’ recovery from acute viral hepatitis despite active maintenance of a cytotoxic T-lymphocyte response. Nat Med. 1996;2(10):1104–1108. 8837608 10.1038/nm1096-1104

[4] Shi Y, Zheng M. Hepatitis B virus persistence and reactivation. Br Med J. 2020 Sep 1;370:m2200. 32873599 10.1136/bmj.m2200

[5] Alter H, Block T, Brown N, et al. A research agenda for curing chronic hepatitis B virus infection. Hepatology. 2018 Mar;67(3):1127–1131. 28877549 10.1002/hep.29509 PMC5873273

[6] Chang J, Block TM, Guo JT. The innate immune response to hepatitis B virus infection: implications for pathogenesis and therapy. Antiviral Res. 2012 Dec;96(3):405–413. 23072881 10.1016/j.antiviral.2012.10.001

[7] Wieland SF, Chisari FV. Stealth and cunning: hepatitis B and hepatitis C viruses. J Virol. 2005 Aug;79(15):9369–9380. 16014900 10.1128/JVI.79.15.9369-9380.2005 PMC1181548

[8] Niu C, Livingston CM, Li L, et al. The Smc5/6 complex restricts HBV when localized to ND10 without inducing an innate immune response and Is counteracted by the HBV X protein shortly after infection. PLoS One. 2017;12(1):e0169648. 28095508 10.1371/journal.pone.0169648 PMC5240991

[9] Gish RG, Given BD, Lai CL, et al. Chronic hepatitis B: virology, natural history, current management and a glimpse at future opportunities. Antiviral Res. 2015 Sep;121:47–58. 26092643 10.1016/j.antiviral.2015.06.008

[10] Bertoletti A, Ferrari C. Adaptive immunity in HBV infection. J Hepatol. 2016 Apr;64(1 Suppl):S71–S83. 27084039 10.1016/j.jhep.2016.01.026

[11] Guidotti LG, Rochford R, Chung J, et al. Viral clearance without destruction of infected cells during acute HBV infection [In process citation]. Science. 1999;284(5415):825–829. 10221919 10.1126/science.284.5415.825

[12] Lucifora J, Xia Y, Reisinger F, et al. Specific and nonhepatotoxic degradation of nuclear hepatitis B virus cccDNA. Science. 2014 Mar 14;343(6176):1221–1228. 24557838 10.1126/science.1243462 PMC6309542

[13] Wieland SF, Spangenberg HC, Thimme R, et al. Expansion and contraction of the hepatitis B virus transcriptional template in infected chimpanzees. Proc Natl Acad Sci U S A. 2004 Feb 17;101(7):2129–2134. 14764900 10.1073/pnas.0308478100 PMC357063

[14] Chen Z, Diaz G, Pollicino T, et al. Role of humoral immunity against hepatitis B virus core antigen in the pathogenesis of acute liver failure. Proc Natl Acad Sci U S A. 2018 Nov 27;115(48):E11369–E11E78. 30420516 10.1073/pnas.1809028115 PMC6275524

[15] Le Bert N, Gill US, Hong M, et al. Effects of hepatitis B surface antigen on virus-specific and global T cells in patients with chronic HBV infection. Gastroenterology. 2020 Aug;159(2):652–664. 32302614 10.1053/j.gastro.2020.04.019

[16] Salimzadeh L, Le Bert N, Dutertre CA, et al. PD-1 blockade partially recovers dysfunctional virus-specific B cells in chronic hepatitis B infection. J Clin Invest. 2018 Oct 1;128(10):4573–4587. 30084841 10.1172/JCI121957 PMC6159957

[17] Burton AR, Pallett LJ, McCoy LE, et al. Circulating and intrahepatic antiviral B cells are defective in hepatitis B. J Clin Invest. 2018 Oct 1;128(10):4588–4603. 30091725 10.1172/JCI121960 PMC6159997

[18] Boni C, Fisicaro P, Valdatta C, et al. Characterization of hepatitis B virus (HBV)-specific T-cell dysfunction in chronic HBV infection. J Virol. 2007 Apr;81(8):4215–4225. 17287266 10.1128/JVI.02844-06 PMC1866111

[19] Das A, Hoare M, Davies N, et al. Functional skewing of the global CD8 T cell population in chronic hepatitis B virus infection. J Exp Med. 2008 Sep 1;205(9):2111–2124. 18695005 10.1084/jem.20072076 PMC2526205

[20] Lim CJ, Lee YH, Pan L, et al. Multidimensional analyses reveal distinct immune microenvironment in hepatitis B virus-related hepatocellular carcinoma. Gut. 2019 May;68(5):916–927. 29970455 10.1136/gutjnl-2018-316510

[21] Bengsch B, Martin B, Thimme R. Restoration of HBV-specific CD8+ T cell function by PD-1 blockade in inactive carrier patients is linked to T cell differentiation. J Hepatol. 2014 Dec;61(6):1212–1219. 25016223 10.1016/j.jhep.2014.07.005

[22] Li H, Wu K, Tao K, et al. Tim-3/galectin-9 signaling pathway mediates T-cell dysfunction and predicts poor prognosis in patients with hepatitis B virus-associated hepatocellular carcinoma. Hepatology. 2012 Oct;56(4):1342–1351. 22505239 10.1002/hep.25777

[23] Sharpe AH, Pauken KE. The diverse functions of the PD1 inhibitory pathway. Nat Rev Immunol. 2018 Mar;18(3):153–167. 28990585 10.1038/nri.2017.108

[24] Alfei F, Kanev K, Hofmann M, et al. TOX reinforces the phenotype and longevity of exhausted T cells in chronic viral infection. Nature. 2019 Jul;571(7764):265–269. 31207605 10.1038/s41586-019-1326-9

[25] Fisicaro P, Barili V, Montanini B, et al. Targeting mitochondrial dysfunction can restore antiviral activity of exhausted HBV-specific CD8 T cells in chronic hepatitis B. Nat Med. 2017 Mar;23(3):327–336. 28165481 10.1038/nm.4275

[26] Peppa D, Gill US, Reynolds G, et al. Up-regulation of a death receptor renders antiviral T cells susceptible to NK cell-mediated deletion. J Exp Med. 2013 Jan 14;210(1):99–114. 23254287 10.1084/jem.20121172 PMC3549717

[27] Lopes AR, Kellam P, Das A, et al. Bim-mediated deletion of antigen-specific CD8 T cells in patients unable to control HBV infection. J Clin Invest. 2008 May;118(5):1835–1845. 18398508 10.1172/JCI33402 PMC2289792

[28] Pallett LJ, Gill US, Quaglia A, et al. Metabolic regulation of hepatitis B immunopathology by myeloid-derived suppressor cells. Nat Med. 2015 Jun;21(6):591–600. 25962123 10.1038/nm.3856 PMC4458139

[29] Sandalova E, Laccabue D, Boni C, et al. Increased levels of arginase in patients with acute hepatitis B suppress antiviral T cells. Gastroenterology. 2012 Jul;143(1):78–87e3. 22475535 10.1053/j.gastro.2012.03.041

[30] Fisicaro P, Valdatta C, Massari M, et al. Antiviral intrahepatic T-cell responses can be restored by blocking programmed death-1 pathway in chronic hepatitis B. Gastroenterology. 2010 Feb;138(2):682–693. 93 e1-4. 19800335 10.1053/j.gastro.2009.09.052

[31] Fanning GC, Zoulim F, Hou J, et al. Therapeutic strategies for hepatitis B virus infection: towards a cure. Nat Rev Drug Discov. 2019 Nov;18(11):827–844. 31455905 10.1038/s41573-019-0037-0

[32] Gane E, Verdon DJ, Brooks AE, et al. Anti-PD-1 blockade with nivolumab with and without therapeutic vaccination for virally suppressed chronic hepatitis B: a pilot study. J Hepatol. 2019 Nov;71(5):900–907. 31306680 10.1016/j.jhep.2019.06.028

[33] Isogawa M, Chung J, Murata Y, et al. CD40 activation rescues antiviral CD8(+) T cells from PD-1-mediated exhaustion. PLoS Pathog. 2013;9(7):e1003490. 23853599 10.1371/journal.ppat.1003490 PMC3708877

[34] Boni C, Vecchi A, Rossi M, et al. TLR7 agonist Increases responses of hepatitis B virus-specific T cells and natural killer cells in patients with chronic hepatitis B treated with nucleos(T)Ide analogues. Gastroenterology. 2018 May;154(6):1764–77e7. 29378197 10.1053/j.gastro.2018.01.030

[35] Pauken KE, Sammons MA, Odorizzi PM, et al. Epigenetic stability of exhausted T cells limits durability of reinvigoration by PD-1 blockade. Science. 2016 Dec 2;354(6316):1160–1165. 27789795 10.1126/science.aaf2807 PMC5484795

[36] Heim K, Neumann-Haefelin C, Thimme R, et al. Heterogeneity of HBV-specific CD8(+) T-cell failure: implications for immunotherapy. Front Immunol. 2019;10:2240. 31620140 10.3389/fimmu.2019.02240 PMC6763562

[37] Ghoneim HE, Fan Y, Moustaki A, et al. De novo epigenetic programs inhibit PD-1 blockade-mediated T cell rejuvenation. Cell. 2017 Jun 29;170(1):142–157e19. 28648661 10.1016/j.cell.2017.06.007 PMC5568784

[38] Kim JH, Ghosh A, Ayithan N, et al. Circulating serum HBsAg level is a biomarker for HBV-specific T and B cell responses in chronic hepatitis B patients. Sci Rep. 2020 Feb 4;10(1):1835. 32020034 10.1038/s41598-020-58870-2 PMC7000714

[39] Loggi E, Bihl FK, Cursaro C, et al. Virus-specific immune response in HBeAg-negative chronic hepatitis B: relationship with clinical profile and HBsAg serum levels. PLoS One. 2013;8(6):e65327. 23750252 10.1371/journal.pone.0065327 PMC3672146

[40] Boni C, Laccabue D, Lampertico P, et al. Restored function of HBV-specific T cells after long-term effective therapy with nucleos(t)ide analogues. Gastroenterology. 2012 Oct;143(4):963–973e9. 22796241 10.1053/j.gastro.2012.07.014

[41] Huang Q, Zhou B, Cai D, et al. Rapid turnover of hepatitis B virus covalently closed circular DNA indicated by monitoring emergence and reversion of signature-mutation in treated chronic hepatitis B patients. Hepatology. 2020 Jan;73(1):41–52. 32189364 10.1002/hep.31240 PMC7898704

[42] Micco L, Peppa D, Loggi E, et al. Differential boosting of innate and adaptive antiviral responses during pegylated-interferon-alpha therapy of chronic hepatitis B. J Hepatol. 2013 Feb;58(2):225–233. 23046671 10.1016/j.jhep.2012.09.029

[43] Xu X, Shang Q, Chen X, et al. Reversal of B-cell hyperactivation and functional impairment is associated with HBsAg seroconversion in chronic hepatitis B patients. Cell Mol Immunol. 2015 May;12(3):309–316. 25849120 10.1038/cmi.2015.25 PMC4654326

[44] Zhang W, Zhang D, Dou X, et al. Consensus on pegylated interferon alpha in treatment of chronic hepatitis B. J Clin Transl Hepatol. 2018 Mar 28;6(1):1–10. 29577026 10.14218/JCTH.2017.00073 PMC5862993

[45] van den Berg F, Limani SW, Mnyandu N, et al. Advances with RNAi-based therapy for hepatitis B virus infection. Viruses. 2020 Aug 4;12(8):851. 10.3390/v12080851 PMC7472220 32759756

[46] Billioud G, Kruse RL, Carrillo M, et al. In vivo reduction of hepatitis B virus antigenemia and viremia by antisense oligonucleotides. J Hepatol. 2016 Apr;64(4):781–789. 26658683 10.1016/j.jhep.2015.11.032

[47] Mueller H, Wildum S, Luangsay S, et al. A novel orally available small molecule that inhibits hepatitis B virus expression. J Hepatol. 2018 Mar;68(3):412–420. 29079285 10.1016/j.jhep.2017.10.014

[48] Guidotti LG, Matzke B, Schaller H, et al. High-level hepatitis B virus replication in transgenic mice. J Virol. 1995;69(10):6158–6169. 7666518 10.1128/jvi.69.10.6158-6169.1995 PMC189513

[49] McFadden VC, Shalaby RE, Iram S, et al. Hepatic deficiency of the pioneer transcription factor FoxA restricts hepatitis B virus biosynthesis by the developmental regulation of viral DNA methylation. PLoS Pathog. 2017 Feb;13(2):e1006239. 28235042 10.1371/journal.ppat.1006239 PMC5342274

[50] Benechet AP, De Simone G, Di Lucia P, et al. Dynamics and genomic landscape of CD8(+) T cells undergoing hepatic priming. Nature. 2019 Oct;574(7777):200–205. 31582858 10.1038/s41586-019-1620-6 PMC6858885

[51] Thomsen MK, Nandakumar R, Stadler D, et al. Lack of immunological DNA sensing in hepatocytes facilitates hepatitis B virus infection. Hepatology. 2016 Sep;64(3):746–759. 27312012 10.1002/hep.28685

[52] Dion S, Bourgine M, Godon O, et al. Adeno-associated virus-mediated gene transfer leads to persistent hepatitis B virus replication in mice expressing HLA-A2 and HLA-DR1 molecules. J Virol. 2013 May;87(10):5554–5563. 23468504 10.1128/JVI.03134-12 PMC3648192

[53] Qi Z, Li G, Hu H, et al. Recombinant covalently closed circular hepatitis B virus DNA induces prolonged viral persistence in immunocompetent mice. J Virol. 2014 Jul;88(14):8045–8056. 24807718 10.1128/JVI.01024-14 PMC4097776

[54] Fumagalli V, Di Lucia P, Venzin V, et al. Serum HBsAg clearance has minimal impact on CD8+ T cell responses in mouse models of HBV infection. J Exp Med. 2020 Nov 2;217(11):e20200298. 32761167 10.1084/jem.20200298 PMC7596822

[55] Kawashima K, Isogawa M, Onishi M, et al. Restoration of type I interferon signaling in intrahepatically primed CD8+ T cells promotes functional differentiation. JCI Insight. 2021 Feb 8;6(3):e145761. 10.1172/jci.insight.145761 PMC7934883 33400688

[56] Michler T, Kosinska AD, Festag J, et al. Knockdown of virus antigen expression increases therapeutic vaccine efficacy in high-titer hepatitis B virus carrier mice. Gastroenterology. 2020 May;158(6):1762–75e9. 32001321 10.1053/j.gastro.2020.01.032

[57] Zhu D, Liu L, Yang D, et al. Clearing persistent extracellular antigen of hepatitis B virus: an immunomodulatory strategy to reverse tolerance for an effective therapeutic vaccination. J Immunol. 2016 Apr 1;196(7):3079–3087. 26936879 10.4049/jimmunol.1502061 PMC4824405

[58] Menne S, Cote PJ. The woodchuck as an animal model for pathogenesis and therapy of chronic hepatitis B virus infection. World J Gastroenterol. 2007 Jan 7;13(1):104–124. 17206759 10.3748/wjg.v13.i1.104 PMC4065868

[59] Menne S, Tumas DB, Liu KH, et al. Sustained efficacy and seroconversion with the toll-like receptor 7 agonist GS-9620 in the woodchuck model of chronic hepatitis B. J Hepatol. 2015 Jun;62(6):1237–1245. 25559326 10.1016/j.jhep.2014.12.026 PMC4439359

[60] Gane EJ, Lim YS, Gordon SC, et al. The oral toll-like receptor-7 agonist GS-9620 in patients with chronic hepatitis B virus infection. J Hepatol. 2015 Aug;63(2):320–328. 25733157 10.1016/j.jhep.2015.02.037

[61] Lanford RE, Guerra B, Chavez D, et al. GS-9620, an oral agonist of toll-like receptor-7, induces prolonged suppression of hepatitis B virus in chronically infected chimpanzees. Gastroenterology. 2013 Jun;144(7):1508–1517. 17 e1-10. 23415804 10.1053/j.gastro.2013.02.003 PMC3691056

[62] Zhou T, Block T, Liu F, et al. HBsag mRNA degradation induced by a dihydroquinolizinone compound depends on the HBV posttranscriptional regulatory element. Antiviral Res. 2018 Jan;149:191–201. 29133129 10.1016/j.antiviral.2017.11.009

[63] Menne S, Wildum S, Steiner G, et al. Efficacy of an inhibitor of hepatitis B virus expression in combination With entecavir and interferon-alpha in woodchucks chronically infected With woodchuck hepatitis virus. Hepatol Commun. 2020 Jun;4(6):916–931. 32490326 10.1002/hep4.1502 PMC7262289

[64] Wooddell CI, Rozema DB, Hossbach M, et al. Hepatocyte-targeted RNAi therapeutics for the treatment of chronic hepatitis B virus infection. Mol Ther. 2013 May;21(5):973–985. 23439496 10.1038/mt.2013.31 PMC3666629

[65] Wooddell CI, Yuen MF, Chan HL, et al. RNAi-based treatment of chronically infected patients and chimpanzees reveals that integrated hepatitis B virus DNA is a source of HBsAg. Sci Transl Med. 2017 Sep 27;9(409):eaan0241. 28954926 10.1126/scitranslmed.aan0241 PMC5830187

[66] Han K, Cremer J, Elston R, et al. A randomized, double-blind, placebo-controlled, first-time-in-human study to assess the safety, tolerability, and pharmacokinetics of single and multiple ascending doses of GSK3389404 in healthy subjects. Clin Pharmacol Drug Dev. 2019 Aug;8(6):790–801. 30861337 10.1002/cpdd.670 PMC6767536

[67] Vaillant A. Nucleic acid polymers: broad spectrum antiviral activity, antiviral mechanisms and optimization for the treatment of hepatitis B and hepatitis D infection. Antiviral Res. 2016 Jul 9;133:32–40. 27400989 10.1016/j.antiviral.2016.07.004

[68] Noordeen F, Scougall CA, Grosse A, et al. Therapeutic antiviral effect of the Nucleic acid polymer REP 2055 against persistent duck hepatitis B virus infection. PLoS One. 2015;10(11):e0140909. 26560490 10.1371/journal.pone.0140909 PMC4641618

[69] Boulon R, Blanchet M, Lemasson M, et al. Characterization of the antiviral effects of REP 2139 on the HBV lifecycle in vitro. Antiviral Res. 2020 Nov;183:104853. 32585322 10.1016/j.antiviral.2020.104853

[70] Bazinet M, Pantea V, Placinta G, et al. Safety and efficacy of 48 weeks REP 2139 or REP 2165, tenofovir disoproxil, and pegylated interferon alfa-2a in patients with chronic HBV infection naive to nucleos(t)ide therapy. Gastroenterology. 2020 Jun;158(8):2180–2194. 32147484 10.1053/j.gastro.2020.02.058

[71] Corti D, Benigni F, Shouval D. Viral envelope-specific antibodies in chronic hepatitis B virus infection. Curr Opin Virol. 2018 Jun;30:48–57. 29738926 10.1016/j.coviro.2018.04.002

[72] Neumann AU, Phillips S, Levine I, et al. Novel mechanism of antibodies to hepatitis B virus in blocking viral particle release from cells. Hepatology. 2010 Sep;52(3):875–885. 20593455 10.1002/hep.23778 PMC3086357

[73] Li D, He W, Liu X, et al. A potent human neutralizing antibody Fc-dependently reduces established HBV infections. eLife. 2017 Sep 26;6:e26738. 28949917 10.7554/eLife.26738 PMC5614562

[74] Zhang TY, Yuan Q, Zhao JH, et al. Prolonged suppression of HBV in mice by a novel antibody that targets a unique epitope on hepatitis B surface antigen. Gut. 2016 Apr;65(4):658–671. 26423112 10.1136/gutjnl-2014-308964

[75] Hehle V, Beretta M, Bourgine M, et al. Potent human broadly neutralizing antibodies to hepatitis B virus from natural controllers. J Exp Med. 2020 Oct 5;217(10):e20200840. 32579155 10.1084/jem.20200840 PMC7537403

[76] Wang Q, Michailidis E, Yu Y, et al. A combination of human broadly neutralizing antibodies against hepatitis B virus HBsAg with distinct epitopes suppresses escape mutations. Cell Host Microbe. 2020 Aug 12;28(2):335–349e6. 32504577 10.1016/j.chom.2020.05.010 PMC8182833

[77] Shi B, Wu Y, Wang C, et al. Evaluation of antiviral - passive - active immunization (“sandwich”) therapeutic strategy for functional cure of chronic hepatitis B in mice. EBioMedicine. 2019 Nov;49:247–257. 31680000 10.1016/j.ebiom.2019.10.043 PMC6945269

[78] Galun E, Eren R, Safadi R, et al. Clinical evaluation (phase I) of a combination of two human monoclonal antibodies to HBV: safety and antiviral properties. Hepatology. 2002 Mar;35(3):673–679. 11870383 10.1053/jhep.2002.31867

[79] Bournazos S, Corti D, Virgin HW, et al. Fc-optimized antibodies elicit CD8 immunity to viral respiratory infection. Nature. 2020 Dec;588(7838):485–490. 33032297 10.1038/s41586-020-2838-z PMC7672690

[80] Fisicaro P, Barili V, Rossi M, et al. Pathogenetic mechanisms of T cell dysfunction in chronic HBV infection and related therapeutic approaches. Front Immunol. 2020;11:849. 32477347 10.3389/fimmu.2020.00849 PMC7235343

